# Relationship of *APOA5*, *PPARγ* and HL gene variants with serial changes in childhood body mass index and coronary artery disease risk factors in young adulthood

**DOI:** 10.1186/1476-511X-10-68

**Published:** 2011-05-08

**Authors:** Lakshmy Ramakrishnan, Harshpal S Sachdev, Meenakshi Sharma, Ransi Abraham, Swami Prakash, Dileep Gupta, Yogendra Singh, Seema Bhaskar, Shikha Sinha, Giriraj R Chandak, Kolli S Reddy, Bhargava Santosh

**Affiliations:** 1 Department of Cardiac Biochemistry, All India Institute of Medical Sciences, Ansari Nagar, New Delhi-110029, India; 2 Department of Pediatrics and Clinical Epidemiology, Sitaram Bhartia Institute of Science and Research, New Delhi, India; 3 Non Communicable Disease Division, Indian Council of Medical Research, New Delhi, India; 4 Genome Research Group, Centre for Cellular and Molecular Biology, Hyderabad, India; 5 Public Health Foundation of India, New Delhi, India; 6 Department of Pediatrics, Sunder Lal Jain Hospital, New Delhi, India

## Abstract

**Background:**

Triglycerides is an independent risk factor for coronary artery disease (CAD) and is especially important in Indians because of high prevalence of hypertriglyceridemia in this population. Both genetic and environmental factors determine triglyceride levels. In a birth cohort from India, hypertriglyceridemia was found in 41% of men and 11% of women. Subjects who had high triglycerides had more rapid body mass index (BMI) or weight gain than rest of the cohort throughout infancy, childhood and adolescence. We analysed polymorphisms in *APOA5*, hepatic lipase and *PPARγ* genes and investigated their association with birth weight and serial changes in BMI.

**Results:**

Polymorphisms in *APOA5* (-1131T > C, S19W), *PPARγ* (Pro12Ala) and hepatic lipase (-514C > T) were studied by polymerase chain reaction (PCR) followed by restriction digestion in 1492 subjects from the New Delhi Birth Cohort (NDBC). We assessed whether these polymorphisms influence lipid and other variables and serial changes in BMI, both individually and together.

The risk allele of *APOA5* (-1131C) resulted in 23.6 mg/dl higher triglycerides as compared to normal allele (P < 0.001). Risk allele of HL (-514T) was associated with significantly higher HDL2 levels (P = 0.002). Except for the marginal association of *PPARγ* Pro12Ala variation with a lower conditional weight at 6 months, (P = 0.020) and A*POA5* S19W with a higher conditional BMI at 11 yrs of age (P = 0.030), none of the other associations between the gene polymorphisms and serial changes in body mass index from birth to young adulthood were significant.

**Conclusion:**

The promoter polymorphism in *APOA5* was associated with raised serum triglycerides and that of HL with raised HDL2 levels. None of the polymorphisms had any significant relationship with birth weight or serial changes in anthropometry from birth to adulthood in this cohort.

## Background

Interaction between genetic and environmental factors determines susceptibility of an individual to develop coronary artery disease. Triglyceride is an independent risk factor for coronary artery disease (CAD) and is especially important in Indians because of high prevalence of hypertriglyceridemia in this population. In a well-established birth cohort (New Delhi Birth Cohort, NDBC) of individuals born between the years 1969 to 1973 and followed up in many phases in terms of anthropometric measures and in 2002 for biochemical risk factors of diabetes and CVD, we have earlier demonstrated an inverse relation between thinness in infancy and the presence of impaired glucose tolerance or diabetes in 1492 young adults [1]. The New Delhi Birth Cohort was drawn from all families living in a defined are of South Delhi, India between 1969-1973. Among a population of 119,799 there were 20,755 married women of reproductive age who were assessed every other month in order to record menstrual dates. Women who became pregnant were visited every two months initially and alternate days from 37^th ^week of gestation. There were 9169 pregnancies resulting in 8181 live births. Trained personnel recorded the weight and the length of the babies within 72 hrs of birth, at the ages of 3, 6, 9 and 12 months (±7 days) and at 6 months interval thereafter. There were many phases in this cohort study (phase 1: 1969-1973, phase 2: 1974-1980, phase 3: 1983-1987, phase 4: 1987-1991, Current phase: 1998-2002). More than 30% of the cohort was lost between the end of phase I and beginning phase 2, a time when unauthorized housing was demolished in South Delhi. In the current phase, between 1998-2002, 31.6% of the initial cohort could be located. 1526 subjects provided consent and participated. Close to 1/10^th ^of these subjects had impaired glucose tolerance (10.8%), and 4.4% were diabetic. Another important finding was that 41% of men and 11% of women had hyper-triglyceridemia. Subjects who had high triglycerides had more rapid body mass index (BMI) or weight gain than rest of the cohort throughout infancy, childhood and adolescence [2].

Triglyceride metabolism is both under genetic and environmental influences. It is likely that genes which influence triglyceride levels have different effects on people with different birth weight and or weight at infancy or childhood [3]. *APOA5* is an important determinant of triglyceride levels [4] and it's over expression results in decreased plasma triglyceride levels [5]. Two polymorphisms, -1131T > C and S19W, in the *APOA5* gene have been shown to be associated with raised triglyceride levels in different populations [4]. *PPAR-γ* regulates a number of adipocyte genes involved in pathways of lipid metabolism. A "G" to "C" substitution in exon coding for *PPAR-γ* results in proline to alanine amino acid change (*PPARγ* Pro12Ala). The Ala isoform of *PPAR-γ* is less effective at targeting genes and is found to be significantly associated with dyslipidemia in obese [6]. Hepatic lipase hydrolyzes triglycerides from lipoproteins in circulation. Polymorphism in promoter region in the *HL *gene (C-514T) is associated with diminished lipase activity, dyslipidemia, and atherosclerosis [7]. We studied the relative role of two *APOA5* gene polymorphisms: -1131T > C and S19W, *PPARγ* gene polymorphism- Pro12Ala and hepatic lipase C-514T polymorphism on lipid levels and also the relationship with serial changes in BMI from birth to adulthood in the NDBC.

### Subjects and methods

The study was carried out from blood samples of the NDBC subjects (n = 1492) stored at -70°C which were collected during the period 1998 to 2002 (Details published elsewhere [1]). Demographic details available for the subjects included sex, age, residence, socio-economic status, education and employment. Anthropometric measurements including birth weight, length and gestation, serial anthropometric profile from birth till young adulthood (six monthly intervals), and current weight, height, waist, and hip circumferences, skin fold thicknesses (triceps and subscapular) were also available. The methods of estimations have been detailed in the earlier publication [1]. Fresh blood samples were collected for measurement of LDL particle diameter and HDL3 levels. LDL particle diameter was measured by electrophoresis [8]. HDL 3 was measured in serum by dual precipitation method [9]. HDL2 was derived from total HDL and HDL3. The study was approved by the All India Institute of Medical Sciences (AIIMS) research ethics committee and informed consent was taken from all subjects.

### DNA extraction and genotyping

DNA was extracted by salting out method [10]. *APOA5* -1131T > C polymorphism was genotyped by ARMS (Amplification Refractory Mutation System)-PCR method described by Chandak *et al *[11]. The primers amplify a control product of 404 bp while the PCR products of 250 bp and 242 bp identify T and C alleles respectively. The S19W polymorphism in APOA5 was determined by PCR-RFLP amplifying a 157 bp product. To determine *PPAR-γ* Pro12Ala polymorphism, mutagenically separated PCR was performed. A 230 bp product identifies the Pro allele and 250 bp product the Ala allele. To assay the C-514T HL polymorphism in the promoter region, a 299-bp fragment containing the restriction site was PCR-amplified. The amplified DNA was digested with *Nla III* generating two fragments of 229 bp and 70 bp in subjects with "T" allele. Positive and negative controls were included in all RFLP runs. For quality assurance of data generated, 10% samples were regenotyped at CCMB and a near 100% concordance was observed. This validates the data generated on all the samples and hence the association analyses conducted on the genotypic and allelic frequency is robust

### Statistical methods

All biochemical parameters were analyzed for normality. Appropriate transformations were required for skewed variables prior to analyses. Allele frequencies were computed by allele counting. Concordance of genotype frequencies with Hardy Weinberg equilibrium (HWE) was tested with χ2 goodness of fit. We investigated the relationship between genotype and all biochemical and clinical variables by ANOVA and the linear differences in genotype associations by trend test. These genotypic associations were also tested with the dominant and recessive models. We generated weight, height and BMI internal sex-specific standard deviation (SD) scores. We modeled the progress of the median, spread and skewness of the measurements as age increased. For each subject we interpolated values linearly between successive SD scores to estimate SD scores at 6 months and at birthdays from 1 to 21 years. The interpolated values were used if measurements were made within 6 months (up to 1 year), 1 year (age 2 years), 1.5 years (age 3 years) and 2 years (all older ages). Back-transformation provided estimates of measurements at these ages. In order to measure changes in early-life BMI and height (growth), we used the conditional SD scores method. We divided growth into three periods: birth to 2 years (infancy), 2-11 years (pre-pubertal childhood growth) and 11 years to adulthood (adolescent growth). To describe growth during each interval, for example between 2 and 11 years, we regressed SD scores at the end of the interval (11 years) on SD scores at the beginning (2 years) and at all preceding time points (birth, 6 months, 1 year), and expressed the residuals as SD scores. This produces uncorrelated variables describing change between specific ages (conditional SD scores). We also conducted multiple linear regression analyses for evaluating the associations after adjustment for confounders.

## Results

### Frequency of -1131T > C and S19W in *APOA5*, C-514T in hepatic lipase and Pro12Ala in *PPAR-γ* polymorphisms in the NDBC cohort

The allelic and genotype frequencies of the four evaluated SNPs are depicted in Table 1. The risk allele frequency of hepatic lipase (C-514T) was highest (22.9%), then *APOA5* (-1131T > C) (18.7%), followed by *PPAR-γ* (Pro 12Ala) (11.2%). *APOA5* (S19W) minor allele was rare (4.0%). The genotype frequencies at all the polymorphisms did not deviate from the Hardy-Weinberg equilibrium (p > 0.05).

**Table 1:** Frequency (%) of single nucleotide polymorphisms (SNP) in Subjects

**SNP**	**N**	**Wild Homozygous N (%)**	**Heterozygous N (%)**	**Mutant Homozygous N (%)**	**Minor Allele frequency**	P value*
Hepatic lipase (-514C > T)	1326	797 (60.1)	451 (34.0)	78 (5.9)	22.9	0.414

*PPAR-γ* (Pro12Ala) rs1801282	1219	970 (79.6)	231 (18.9)	18 (1.5)	11.0	0.611

*APOA5* (-1131T > C) rs662799	1226	811 (66.2)	372 (30.3)	43 (3.5)	18.7	0.999

*APOA5* (S19W) rs3135506	1140	1053 (92.4)	84 (7.4)	3 (0.3)	4.0	0.633

* P value for Hardy-Weinberg equilibrium

### Inter-relationship between gene variants and coronary artery disease risk factors in adults

The inter-relationship between the four polymorphisms and lipid variables for both sexes combined is depicted in Table 2. The analyses were also performed for men and women separately (data not given).

**Table 2:** Association of *APOA*5 (-1131T > C, S19W), HL (C-514T) and *PPAR γ *(Pro12Ala) genotypes with lipid variables in adults

**Genotype (n)**	**Cholesterol (mg/dl)**	**Triglycerides (mg/dl)**	**Total HDL (mg/dl)**	**HDL2** (mg/dl)^*^	**LDL** **(mg/dl)**	**LDL particle size (nm)**
***APOA5* (-1131T > C)**						

TT (779)	191.5 ± 41.3	126.9 ± 70.1	47.5 ± 11.4	17.0 (13.0-21.0)	118.4 ± 34.8	27.0 ± 0.6

TC (350)	196.9 ± 41.9	150.5 ± 96.7	46.1 ± 11.7	17.0 (13.0-21.0)	119.6 ± 34.8	26.9 ± 0.6

CC (41)	205.2 ± 43.2	184.4 ± 104.1	46.0 ± 10.5	18.0 (13.5-22.5)	122.3 ± 34.4	27.0 ± 0.6

P value	**0.025**	**<0.0001**	0.132	0.822	0.703	0.760

P value (linear trend)	**0.007**	**<0.0001**	0.055	0.558	0.418	0.550

Deviation from linearity	0.714	0.505	0.542	0.827	0.823	0.661

P value (Dominant Model) (1129/41)	0.068	**<0.0001**	0.562	0.619	0.522	0.978

P value (Recessive Model) (779/391)	**0.015**	**<0.0001**	**0.044**	0.624	0.487	0.480

***APOA5* (S19W)**						

SS (1000)	192.3 ± 41.7	133.2 ± 80.2	46.9 ± 11.5	17.0 (13.0-21.0)	118.5 ± 35.1	27.0 ± 0.6

SW/WW (84)	197.1 ± 41.2	149.9 ± 89.8	45.9 ± 13.1	18.0 (14.0-20.75)	120.7 ± 33.6	26.9 ± 0.7

P value (Dominant Model) (1081/3)	0.324	**0.030**	**0.016**	0.928	0.542	0.417

P value (Recessive Model) (1000/84)	0.312	0.069	0.461	0.742	0.578	0.491

**HL (C-514T)**						

CC (756)	191.8 ± 40.0	133.7 ± 80.8	47.1 ± 11.6	17.0 (12.0-20.0)	117.6 ± 33.4	27.0 ± 0.6

CT(434)	194.7 ± 43.4	134.6 ± 78.8	47.6 ± 11.7	18.0 (14.0-23.0)	119.7 ± 36.4	27.0 ± 0.6

TT(76)	196.6 ± 48.6	159.6 ± 100.0	46.3 ± 12.3	18.0 (13.0-23.0)	118.2 ± 38.6	26.9 ± 0.5

P value	0.404	**0.029**	0.591	**0.002**	0.604	0.090

P value (linear trend)	0.180	0.065	0.960	**0.002**	0.460	0.921

Deviation from linearity	0.893	0.056	0.306	0.098	0.496	**0.028**

P value (Dominant Model) (1190/76)	0.451	**0.008**	0.450	0.451	0.958	0.106

P value (Recessive Model) (756/510)	0.196	0.318	0.669	**<0.0001**	0.348	0.367

***PPARγ* (Pro12Ala)**						

Pro12Pro (916)	193.0 ± 41.6	136.2 ± 82.4	47.1 ± 11.8	17.0 (13.0-21.0)	118.1 ± 34.2	27.0 ± 0.7

Pro12Ala/Ala12Ala (245)	195.4 ± 44.3	138.7 ± 80.7	47.7 ± 11.4	17.0 (14.0-21.75)	119.8 ± 38.7	27.0 ± 0.6

P value (Dominant Model) (1143/18)	0.136	**0.029**	0.820	0.110	0.415	0.493

P value (Recessive Model) (916/245)	0.437	0.671	0.473	0.173	0.496	0.530

Values are mean ± SD

* Transformed (square root) values were used.

A significant difference in triglycerides was evident with subjects having "CC" genotype showing the highest mean triglyceride levels (p < 0.0001 for linear trend). This association remained significant when males (p < 0.001) and females (p = 0.046) were analyzed separately. The age, sex and BMI adjusted effect size per allele of the linear association between *APOA5* (-1131T > C) SNP and serum triglyceride level was considerable (23.6 mg/dl, 95% CI 16.0 to 31.2). Serum cholesterol was also significantly higher in subjects with CC genotype (p = 0.007 for linear trend). Associations with other lipid variables were not significant. In view of the low minor allele frequency (MAF) of *APOA5* S19W, a combined analysis of heterozygous (SW) and homozygous subjects for W allele (WW) was considered to be more pertinent. Although triglyceride concentration tended to be higher and HDL concentration lower in subjects with minor allele, the differences were not statistically significant by recessive model (p = 0.069 and p = 0.461, respectively). No consistent significant associations were observed between *APOA5* (S19W) polymorphism and any of the other evaluated outcome measures.

For HL (C-514T), HDL2 was higher in subjects with the minor allele (p = 0.002 by linear trend and p < 0.0001 by recessive model). This association remained significant in males when analyzed separately whereas it was weaker in females (p = 0.011). The significant association between C-514T variation in hepatic lipase and HDL2 subfraction persisted even after adjustment for age, gender and BMI (p = 0.002). There was a significant association of minor allele with serum triglyceride (p = 0.029 and p = 0.008 dominant model). No consistent significant associations were observed between polymorphism at *PPAR-γ* (Pro 12Ala) and any of the evaluated outcome measures. The significant association with serum triglyceride in dominant model (p = 0.029) was not evident in recessive model (p = 0.671).

There was no significant association with any of the four polymorphisms and the presence of hypertension (except for *PPAR-γ*) and impaired glucose tolerance or diabetes (Table 3). A borderline statistically significant association was documented between the *PPAR-γ* (Pro 12Ala) SNP and hypertension (p = 0.042).

**Table 3:** Prevalence of IGT/Diabetes and Hypertension in relation to genotype

**SNP**		**IGT/Diabetes present %(n)**	**P value**	**Hypertension present %(n)**	**P value**
***APOA5* (-1131T >C)** **(Dominant Model)**	TT & TC	15.2 (1093)	0.168	9.6 (1132)	0.387
	
	CC	22.0 (41)		11.9 (42)	

***APOA5* (-1131T >C)** **(Recessive Model)**	TT	14.9 (753)	0.258	10.4 (778)	0.151
	
	TC &CC	16.5(381)		8.3 (396)	

***APOA5* (S19W)** **(Dominant Model)**	SS & SW	15.3 (1044)	0.717	8.9 (1087)	0.246
	
	WW	0.0 (2)		33.3 (3)	

***APOA5* (S19W)** **(Recessive Model)**	SS	15.6 (967)	0.203	8.8 (1008)	0.312
	
	SW&WW	11.4 (79)		11.0 (82)	

**HL (C-514T)** **(Dominant Model)**	CC & CT	15.6 (1151)	0.233	9.8 (1192)	0.480
	
	TT	19.4 (72)		10.5 (76)	

**HL (C-514T)** **(Recessive Model)**	CC	14.3 (732)	0.055	9.5 (761)	0.313
	
	CT&TT	17.9 (491)		10.5 (507)	

***PPARγ* (Pro12Ala)** **(Dominant Model)**	Pro12Pro & Pro12Ala	15.5 (1104)	0.546	9.6 (1148)	0.476
	
	Ala12Ala	16.7 (18)		5.6 (18)	

***PPARγ* (Pro12Ala)** **(Recessive Model)**	Pro12Pro	15.2 (884)	0.297	8.7 (921)	**0.042**
	
	Pro12Ala & Ala12Ala	16.8 (238)		12.7 (245)	

### Inter-relationship between gene variants and serial changes in body mass index and weight from birth to young adulthood

Participants had a mean (SD) of 23 (5.5) observations between birth and the age of 21 years. Association between BMI at birth and conditional BMI at later ages and gene variants is given in Table 4. Associations between birth weight and conditional weight at later ages were also analyzed (data not shown). In view of sample size restrictions, the recessive model may be a more meaningful assessment of the relationship. With the recessive model, except for two, none of the other associations between the gene polymorphisms and serial changes in body mass index or weight from birth to young adulthood were statistically significant. PPAR gamma Pro12Ala variation was associated with a lower conditional weight (p = 0.020) at 6 months (data not shown) and *APOA5* S19W with a higher conditional BMI at 11 yrs (p = 0.030), With the dominant model, except for three, no other associations were significant. The APOA5 (-1131T > C) minor allele was significantly associated with a higher BMI at birth (p = 0.001) and lower conditional BMI at 2 years (p = 0.05). HL (C-514T) minor allele was significantly associated with lower conditional BMI at 2 years (p = 0.034).

**Table 4:** Inter-relationship between polymorphisms at -1131T >C of *APOA5*, S19W of *APOA5*, C-514T of Hepatic lipase and Pro12Ala of PPARγ with BMI at birth and conditional BMI at 6 months, 2 yrs, 11 yrs and adulthood

**Conditional BMI** **(n)**	**at birth** **Mean ± SD**	**6 months** **Mean ± SD**	**2 Yrs** **Mean ± SD**	**11Yrs** **Mean ± SD**	**Adult** **Mean ± SD**
***APOA5* -1131T > C**					

**TT**	0.006 ± 0.99	-0.008 ± 0.96	0.007 ± 0.97	-0.019 ± 0.99	0.036 ± 0.96

**TC/CC**	0.024 ± 1.03	-0.014 ± 1.02	-0.011 ± 0.99	0.053 ± 1.02	-0.003 ± 1.04

**P value (Dominant Model) (846/34)**	**0.001**	0.830	**0.050**	0.242	0.697

**P value (Recessive Model) (592/288)**	0.803	0.935	0.802	0.312	0.578

***APOA5* S19W**					

**SS**	0.039 ± 1.00	0.003 ± 0.98	0.014 ± 1.00	-0.019 ± 0.98	0.012 ± 0.99

**SW/WW**	-0.071 ± 0.92	0.146 ± 0.96	0.003 ± 1.02	0.261 ± 1.01	-0.038 ± 1.05

**P value (Dominant Model) (833/2)**	0.848	0.185	0.835	0.551	0.173

**P value (Recessive Model) (772/63)**	0.397	0.270	0.937	**0.030**	0.700

***HL *(C-514T)**					

**CC**	-0.004 ± 1.02	-0.031 ± 1.00	0.020 ± 1.01	-0.043 ± 0.98	0.032 ± 1.01

**CT/TT**	0.030 ± 0.98	0.004 ± 1.01	-0.050 ± 1.00	0.036 ± 1.00	0.002 ± 0.96

**P value (Dominant Model) (897/59)**	0.854	0.229	**0.034**	0.751	0.821

**P value (Recessive Model) (581/375)**	0.610	0.598	0.295	0.225	0.645

***PPARγ* (Pro12Ala)**					

**Pro12Pro (730)**	-0.014 ± 0.98	0.004 ± 1.00	0.025 ± 0.98	-0.002 ± 1.00	0.018 ± 0.97

**Pro12Ala/Ala12Ala (186)**	0.058 ± 1.10	-0.151 ± 0.94	-0.074 ± 1.04	-0.026 ± 0.99	0.033 ± 1.05

**P value (Dominant Model) (863/14)**	0.991	0.164	0.108	0.791	0.945

**P value (Recessive Model) (703/174)**	0.394	0.065	0.241	0.782	0.853

### Interaction between APOA5 -1131T > C and hepatic lipase C-514T for serum triglyceride

Amongst 1169 participants, 153 (13.1%) subjects had a minor allele of both APOA5 -1131T > C and *HL *C-514T. There was evidence of a significant negative (p = 0.022) interaction between -1131T > C polymorphism in *APOA5* and C-514T polymorphism in hepatic lipase for their age, sex and BMI adjusted association with serum triglyceride levels (Table 5). In the presence of a normal allele of *APOA5* or hepatic lipase, the presence of minor allele in the other gene was associated with a significant elevation in serum triglyceride level (10.5 mg/dl for hepatic lipase and 29 mg/dl for *APOA5*). However, the combination of a minor allele of *APOA5* or hepatic lipase with the minor allele of the other gene (hepatic lipase or *APOA5*) was associated with a decline (not statistically significant) in serum triglyceride level (Table 5). Thus while considering both the mutations simultaneously, as the genotype shifts from wild type to risk one, the association becomes negative.

**Table 5:** Age, Sex and BMI adjusted interaction between hepatic lipase and *APOA5 and *triglyceride levels

	**Serum triglycerides**		
**Model**	**B**	**95% CI**	**P**
Constant	112.74	-69.35, 294.82	0.225

Adult age (yrs)	-2.47	-7.32, -2.39	0.318

Sex	-51.92	-62.39, -41.45	0.000

BMI	2.21	1.16, 3.27	0.000

*APOA5* -1131T > C	56.47	23.88, 89.07	0.001

*HL *C-514T	40.31	8.95, 71.67	0.012

*APOA5/HL*	-25.91	-48.00, -3.82	0.022

Dependent Variable: Serum triglyceride (mg/dl)

APOA5/*HL *represents the interaction term between *APOA5* -1131T > C and C-514T Hepatic Lipase.

## Discussion

To the best of our knowledge no study has evaluated the relationship of polymorphisms in *APOA5*, hepatic lipase and *PPARγ* and serial changes in body mass index from birth to adulthood. In our study, *APOA5* 1131T > C minor allele was associated with significantly higher triglycerides and that of *HL *C-514T was associated with higher HDL2. PPAR gamma Pro12Ala variation was associated with a lower conditional weight at 6 months in heterozygous subjects and those homozygous for minor allele. *APOA5* S19W was associated with a higher conditional BMI at 11 yrs in heterozygous subjects and those homozygous for minor allele. None of the other associations between the gene polymorphisms and serial changes in body mass index or weight from birth to young adulthood were statistically significant.

The minor allele frequency of *APOA5* -1131T > C was more prevalent than the S19W polymorphism in the NDBC (18.7% vs. 4%, respectively). Chandak et al [11] reported a comparable frequency of less common allele of -1131T > C polymorphism in the promoter region of *APOA5* in 20% and that of S19W in 3% in Pune Indians whereas the allele frequency for both SNPs in UK white subjects were 4% and 6%. The reported prevalence of -1131C allele from other parts of the world varies between 6.4% in American Caucasian males [12] and 34% in the Japanese population [13], whilst the 19W allele frequency varies between 0.1% in a Chinese population [14] and 15.8% in Hispanic males [11].

We observed that the presence of the -1131C allele was associated with 23.6 mg/dl higher fasting triglyceride concentrations and 9.9 mg/dl higher post-prandial triglycerides. The reported effect size on triglyceride concentration of the less common "C" allele varies from about 30% in Caucasians [15] to 36% in Chinese [16] and 60% in Turks [17].

We observed a minor allele frequency of 22.9% for C-514T polymorphism in hepatic lipase. The minor allele frequency of 0.15-0.21 has been reported among Caucasians, 0.45-0.53 among Africans and 0.47 among Japanese suggesting considerable ethnic differences. However, a meta-analysis of 25 studies involving nearly 25,000 subjects reported an overall T allele frequency of 25.3% [18]. Plasma HDL and cholesterol differed significantly among genotypes, while LDL and triglycerides levels were similar. To the best of our knowledge there have been no studies on hepatic lipase polymorphisms and their effect on lipids in Indians. We found a significant increase in total HDL and HDL2 levels but not HDL3 levels in carriers of T allele which is on similar lines as reported previously [19-22]. HL is responsible for the conversion of large HDL2 to smaller HDL3 particles by modulating phospholipids content of these particles. A lower HL activity in T carriers will therefore result in higher levels of HDL2. There was a weak association between presence of T allele and triglycerides with the carriers showing a trend towards higher triglycerides in the present study, which is similar to the results obtained in the meta-analysis [18].

Since the only significant effect we found were that of *APOA5* and hepatic lipase on triglycerides and HDL2 sub-fraction, we investigated the interaction between these SNPs in determining triglyceride levels. It was interesting to note the attenuation of the effect of *APOA5* variant on triglycerides in the presence of polymorphism in hepatic lipase and vice versa. Mutation in either of the gene in the presence of a normal genotype in the other gene was associated with higher triglycerides as compared to mutation in both the genes. We could not assess the dietary fat intake, which may interact with HL and *APOA5* polymorphisms to determine APOA5 and HL activity.

We did not find any interaction between birth weight or early growth and the analyzed polymorphisms in determining lipid levels in later life. Ruiz et al [6] studied the influence of *APOE, APOC3* and *PPARγ2* gene polymorphisms on lipid levels in people with low birth weight. Low birth weight was associated with higher total cholesterol, LDL cholesterol, and apoB/apoA in males with *APOE* ε3 ε4 genotype whereas in males the genotype was associated with lower HDL and higher triglycerides. There were no associations between low birth weight and blood lipids in any *PPAR-γ2* genotypes. Effect of *APOE *genotype on total cholesterol, LDL and ApoB has also been reported by others in children with low birth weight [23,24]. The authors suggested that changes in ApoE gene expression may be programmed by *in utero *nutritional events. No group has examined influence of *APOA5* polymorphism on association between birth weight and blood lipids till date. We failed to find any interaction between *APOA5* and birth weight on lipids in our study. Limitation of the NDBC study is that we have not captured current dietary intake and current physical activity which may have an important bearing on lipid levels.

We did not find any significant interaction between birth weight and *PPAR-γ* Pro12Ala genotype on lipid levels. Data on interaction of birth weight and PPAR gamma in determining lipids has been inconclusive with one group underlying the influence of *PPAR-γ* genotypes on the association between birth weight and lipid levels among elderly people [25] and others not finding any significant interaction of birth weight on effect of *PPAR-γ2* genotype on lipids [3,26]. These associations have been linked to developmental plasticity whereby one genotype can give rise to different phenotypes depending on conditions during development.

In conclusion, our study shows that polymorphism in *APOA5, PPAR-γ* and *HL *do not have any significant relationship with the birth weight and serial changes in anthropometry from birth to adulthood. The promoter polymorphism in *APOA5* is associated with a raised serum triglyceride levels; the age, gender and BMI adjusted effect size being substantial. The promoter polymorphism in hepatic lipase is associated with higher HDL2 levels. An interaction between polymorphisms in *APOA5* and hepatic lipase seems to influence the serum triglyceride levels which need to be further explored.

## Competing interests

The authors declare that they have no competing interests.

## Authors' contributions

RL wrote the first draft of the manuscript and was responsible for the analysis. HPS, MS and KSR and SB helped design the study. GRC helped in study design and writing of manuscript. RA performed the gene analysis. SP and SB performed repeat analysis of SNP's for quality assurance, DG and SS carried out the statistical analysis, YS carried out the biochemical analysis. All authors helped in conduct of the study, contributed to the redrafting of the manuscript, and approved the final manuscript.
